# Supplementary material for: Geographic distribution of conversion therapy prevalence in Canada. Findings from a national cross-sectional survey, 2020
Source: F1000Res. 2024 Aug 15;12:844. Originally published 2023 Jul 18. [Version 2] doi: 10.12688/f1000research.134292.2 (PMC10905130; doi:10.12688/f1000research.134292.2)
Supplement: Peer review history

# Reviewer report: Reviewer response for version 2

**DOI:** 10.5256/f1000research.170215.r314339

**Published:** 2024-08-26

**Authors:** Sean Mulcahy

**Affiliations:** 1 La Trobe University, Victoria, Australia

The authors have well addressed the points raised in the initial peer review. In particular, the information on municipal bans is very helpful and could portend an interesting way forward in jurisdictions where federal and provincial legislative bans are not politically feasible.

 There are still some limitations to this study that bear noting in addition to those already identified. Namely, the sample size is very small, being based on a study of 31 participants (though understanding the difficulty of attracting participants research of this kind), and a very proactive cohort making on average 4 reports of conversion therapy practices each (and up to 31). Another limitation of the survey method is that people have different conceptual understandings of what amounts to a conversion therapy practice and may report practices that do not amount to conversion therapy. 

 Obviously, further studies are needed to determine whether conversion practices are geographically specific, as flagged by the authors. Future studies could also usefully explore whether there have been any court judgments against conversion practices and the range of sentences that have been imposed.

Is the work clearly and accurately presented and does it cite the current literature?

Yes

If applicable, is the statistical analysis and its interpretation appropriate?

Yes

Are all the source data underlying the results available to ensure full reproducibility?

Yes

Is the study design appropriate and is the work technically sound?

Yes

Are the conclusions drawn adequately supported by the results?

Yes

Are sufficient details of methods and analysis provided to allow replication by others?

Yes

Reviewer Expertise:

Human rights and law reform for LGBTIQA+ communities.

I confirm that I have read this submission and believe that I have an appropriate level of expertise to confirm that it is of an acceptable scientific standard.

# Reviewer report: Reviewer response for version 1

**DOI:** 10.5256/f1000research.147338.r234548

**Published:** 2024-02-20

**Authors:** Travis Scheadler

**Affiliations:** 1 The Ohio State University, Columbus, Ohio, USA

This study aimed to estimate the spatial patterning of conversion therapy practices (CTPs) in Canada. Overall, the study is well-written and has important policy and research implications. There are a few notes (see below) that should be addressed to improve the quality of the manuscript. These are all relatively minor. 

 Please add a sentence clarifying what you mean by "tip of the iceberg." I understand this is minor, but can help ensure readers unfamiliar with CTPs understand exactly what is being conveyed. 

 Please make sure there is consistency in verb tense. The first sentence says "CTPs are" and the second says "It has" - determine whether CTPs are/is plural or singular. Be sure to check for consistency throughout the manuscript. 

 The first paragraph under "The harms of conversion therapy" is excellent! This is very succinct and straightforward. Great job!

 Consider revising the first sentence in the second paragraph under "Attempts to ban conversion therapy." Changing "are widely discredited and denounced practices" to "have been banned in many parts of Canada" will smooth the transition between these paragraphs and help narrow down the problem to the specific purpose of this study. The beginning of this sentence currently seems more connected to the first half of the Introduction on the generalized harms of CTPs rather than the legislative bans in Canada. 

 Change was to were in the second sentence of the first paragraph under Measures. 

 Table 2 would be less wordy if columns 1 and 2 are switched. For instance, merge all the cells for Alberta to avoid repeating "Alberta"  11 times. 

 Change "conversion practices" to CTPs in the sentence that starts with "The following provinces/territories had <5 reports of conversion practices." 

 The figures are all great visualizations!

 Delete or reword "lifetime and between the years 2010 and 2015, by US state" in the second paragraph of the Discussion. This part of the sentence is cumbersome and confusing to read. Also, in the following sentence, change "the findings from this study" to "the authors." Some readers may mistake "findings from this study" as findings from YOUR current study. Please also consider changing "continue to occur in every US state as recently as the period 2010 to 2015" to "continued to occur in every US state between 2010 and 2015." Then, consider the following revision for the last part of the sentence: "even though major US medical organizations have identified PACGI as ineffective and 20 states and more than 100 municipalities throughout the US have banned PACGI." These changes should improve the readability of this paragraph by offering more consistency in the different lists provided. Another note - 22 states have now banned licensed professionals from providing conversion therapy to minors. Additionally, please be sure to cite the correct sources. The 24th reference is related to the population size of Canada and is unrelated to PACGI in the United States. Be sure to check all references to make sure they are aligned. Note: this is why I marked "partly" to the first question about whether current literature is cited. 

 In the final paragraph before limitations - be careful not to undersell yourself. I appreciate the clarity around how to interpret the findings, but I also think there is room here for a call to action. Consider adding a paragraph - or even just a sentence or two - calling for bans against CTPs in the areas where it is most prevalent. I see that this was briefly mentioned in the Conclusion. I think there is room for it here as well. 

 Add a general sentence to start the limitations section. This can simply be "the present study is not without limitations" or some similar version of this. For the third limitation, add "especially given that some participants were recruited through word-of-mouth." This should help clarify a little more why this is a limitation. 

 Delete the term "underscore" in the second sentence under future research.

Is the work clearly and accurately presented and does it cite the current literature?

Partly

If applicable, is the statistical analysis and its interpretation appropriate?

Yes

Are all the source data underlying the results available to ensure full reproducibility?

Yes

Is the study design appropriate and is the work technically sound?

Yes

Are the conclusions drawn adequately supported by the results?

Yes

Are sufficient details of methods and analysis provided to allow replication by others?

Yes

Reviewer Expertise:

sport for social justice, LGBTQ+ activism and health, LGBTQ+ policies

I confirm that I have read this submission and believe that I have an appropriate level of expertise to confirm that it is of an acceptable scientific standard, however I have significant reservations, as outlined above.

# Response

**Published:** 2024-08-08

**Authors:** Amrit Tiwana

**Affiliations:** Faculty of Health Sciences, Simon Fraser University, Burnaby, British Columbia, Canada

To clarify what we mean by “tip of the iceberg”, we have included a detailed version of the previous sentence on page 3: “CTPs are typically organized (i.e., structured activities) and circumscribed (e.g., a certain number of sessions with a practitioner); these practices are, metaphorically, only the “tip of the iceberg”, representing visible and formally recognized forms of efforts to change LGBTQ2S+ identities.”

 Thank you for pointing out the inconsistency in verb tense. We have reviewed the manuscript and ensured that the subject "CTPs" is consistently treated as a plural noun throughout. We have updated the verb tense to maintain consistency, so all instances now read "CTPs are" or "CTPs have."

 We have changed the sentence accordingly so that it is more connected to the legislative bans in Canada rather than the first half of the introduction on the generalized harms of CTPs. This can be found on page 5: “Given that CTPs have been banned in many parts of Canada, many conversion therapy providers do not advertise themselves as “conversion therapists”.  

 We have changed “was” to “were” in the second sentence of the first paragraph under Measures.

 Thank you for this suggestion regarding the format of Table 2. We discussed it as a team of authors and decided the current ordering—by date of enactment—makes it easy for the reader to see how the cumulative protections accrued over time; therefore, we have left the table as is.

 We have changed “conversion practices” to “CTPs” on page 12: “The following provinces/territories had <5 reports of CTPs and therefore are not further interpreted: Yukon (55.75), PEI (7.00), and Newfoundland and Labrador (1.92). No data were reported regarding the prevalence of ongoing CTPs in the Northwest Territories and Nunavut.”

 We have revised the second paragraph of the Discussion section to improve clarity and readability. We removed the phrase "lifetime and between the years 2010 and 2015, by US state" and rephrased it to better convey the intended meaning. Additionally, we replaced "the findings from this study" with "the authors" to avoid any potential confusion. The sentence now reads: "The authors explored this issue between 2010 and 2015 across US states, finding that PACGI continued to occur in every state between 2010 and 2015, even though major US medical organizations have identified PACGI as ineffective and 22 states and more than 100 municipalities throughout the US have banned PACGI." We also corrected the reference citations to ensure accuracy and alignment with the content. Thank you for this feedback.

 We have incorporated your feedback using a few sentences in the last paragraph for the limitations. This can be found on page 13: “Despite this, our study emphasizes the urgent need for nationwide legislative bans against CTPs in Canada. Areas without bans continue to experience significantly higher prevalence rates, highlighting the ongoing harm inflicted on LGBTQ2S+ individuals. Comprehensive bans, along with rigorous enforcement and support for affected communities, are essential steps toward mitigating these harmful practices and promoting inclusive and affirming environments nationwide.”

 We have added a sentence at the beginning of the paragraph on the limitations. This can be found on page 13: “The present study is not without limitations.”

 The word “underscore” has been deleted. The sentence is now changed to “The results of this study should be confirmed and repeated with a larger sample size to ensure representativeness."

# Reviewer report: Reviewer response for version 1

**DOI:** 10.5256/f1000research.147338.r234559

**Published:** 2024-02-09

**Authors:** Benjamin Harrell

**Affiliations:** 1 Econ, Trinity University, San Antonio, Texas, USA

**Summary: **The study presents the results of a non-probabilistic survey of Canadian adults who either (a) have experienced conversion therapy practices (CTPs) or (b) know someone who has. Importantly, this study generates key knowledge about the geospatial distribution of CTPs across Canada. This is important context because not all territories and provinces ban CTPs, leaving important gaps in legal protections for sexual and gender minorities (SGM). The findings are stark: Only 55.1% of Canadians enjoy protections from CTP, with the highest prevalence of CTPs occurring in the west and the Atlantic regions. Similarly, the findings suggest that protections are imperfectly binding, these practices are still occurring in regions which ban them, albeit at much lower rates--in regions without bans, CTPs occur at 1.76 times the rate of regions WITH bans.

**Assessment: **This important study provides important context to the growing global literature around the prevalence, consequences, and harms of CTPs and other forms of sexual orientation and gender identity change efforts (SOGICE). The study also comes with key limitations. The most immediate limitation of the study is that statistics are derived from a small sample size. Prevalence rates could be biased by the relatively young sample (about 3/4 are younger than 50) and non-representativeness of provincial or territorial residence (there were no responses from Saskatchewan Manitoba, or the territories), Similarly, the sampling strategy (mostly Vancouver, BC based community organizations or CTP survivors) could bias the estimates in ways that are not clear. It could be the case that this survey underestimates the prevalence of CTP due to its under-representativeness of rural SGM, but it could also overestimate the prevalence of CTP due to its focus on survivors of CTP, especially since CTP is known to be correlated with suicidality.

 Despite these limitations, which are both well-documented by authors, and contextualized as directions for future research, this study is a significant contribution to a fundamental question in this research space: WHERE is CTP happening, as much as to whom.

**A note on data availability**: While there is no data made available for replication of this study, the authors make a compelling case for non-inclusion. The data is inherently sensitive and many participants did not provide consent to share their data. However, the authors do provide instructions on how to acquire aggregated data sufficient to qualitatively replicate this study.

Is the work clearly and accurately presented and does it cite the current literature?

Yes

If applicable, is the statistical analysis and its interpretation appropriate?

Yes

Are all the source data underlying the results available to ensure full reproducibility?

No source data required

Is the study design appropriate and is the work technically sound?

Yes

Are the conclusions drawn adequately supported by the results?

Yes

Are sufficient details of methods and analysis provided to allow replication by others?

Yes

Reviewer Expertise:

I am an economist studying sexual and gender minority population health. I specialize in mental healthcare, health policy, and the demography of SGM people. In relation to this paper, I have presented work on the effects of CTP bans in the United States and their effects on suicidality and mental health.

I confirm that I have read this submission and believe that I have an appropriate level of expertise to confirm that it is of an acceptable scientific standard.

# Reviewer report: Reviewer response for version 1

**DOI:** 10.5256/f1000research.147338.r211737

**Published:** 2023-10-04

**Authors:** Sean Mulcahy

**Affiliations:** 1 La Trobe University, Victoria, Australia

This is important research on the geographic distribution of conversion therapy practices in Canadian provinces and territories with and without a ban on these practices.

 The article could be strengthened by addressing the following points.

**First**, what is the nature of bans in those jurisdictions which have them? For example, do they extend to trans/two spirit communities (as the Manitoba non-legislative ban appeared to only extend to sexual orientation)? Are they only focussed on clinical settings (for example, precluding conversations between a child and a parent)?

**Second**, what available information is there on enforcement of bans? For example, are there crime statistics or sentencing data available?

**Third**, what is the effect of municipal (as opposed to provincial or territorial) bans? In this respect, it would be useful to include a full list of municipalities that have enacted legislative bans (and the provinces or territories they are in), particularly given the research found that there were conversion therapy practices in, on average, about 7 municipalities in each province or territory identified.

**Fourth**, whilst the study lists limitations, some of these should be extended as noted. Per limitations 1 and 2, the sample size was indeed very small, being based on a study of 31 participants (though understanding the difficulty of attracting participants research of this kind), and a very proactive cohort making on average 4 reports of conversion therapy practices each (and up to 31). Per limitation 4, another limitation of the survey method is that people have different conceptual understandings of what amounts to a conversion therapy practice and may report practices that do not amount to conversion therapy. Some further limitations also need to be noted: (a) as the study states earlier, "it was not possible to determine whether the... reports were mutually exclusive" meaning that the same practice could have been reported multiple times thus over-emphasising the prevalence of conversion therapy practices in a particular province or territory; (b) conclusions about Prince Edward Island cannot be reached because it was excluded (as one of the "provinces/territories [that] had <5 reports of conversion practices and therefore are not further interpreted").

**Fifth** and finally, the study notes that "results from SN2019 did not find a difference in the prevalence of CTPs across provinces and territories in Canada." Obviously, this study challenges that finding, so it would be useful to articulate why SN2019 did not find a geographic difference and what the possible limitations of that study could be compared to this.

 The study ends with strong conclusions and possible directions for future research, proving the utility and importance of research of this kind.

Is the work clearly and accurately presented and does it cite the current literature?

Yes

If applicable, is the statistical analysis and its interpretation appropriate?

Yes

Are all the source data underlying the results available to ensure full reproducibility?

Yes

Is the study design appropriate and is the work technically sound?

Yes

Are the conclusions drawn adequately supported by the results?

Yes

Are sufficient details of methods and analysis provided to allow replication by others?

Yes

Reviewer Expertise:

Human rights and law reform for LGBTIQA+ communities.

I confirm that I have read this submission and believe that I have an appropriate level of expertise to confirm that it is of an acceptable scientific standard, however I have significant reservations, as outlined above.

# Response

**Published:** 2024-08-08

**Authors:** Amrit Tiwana

**Affiliations:** Faculty of Health Sciences, Simon Fraser University, Burnaby, British Columbia, Canada

First, we have added the following explanatory text to the Introduction (see page 4): “This federal law defined CTP in relation to both sexual orientation and gender identity. While provincial/territorial bans have predominantly focused on clinical settings, the federal law applies to all settings, including those outside licensed healthcare. However, informal practices like parent-child conversations are typically not covered by these bans. Nonetheless, legislation represents an important milestone in the government’s commitment to protecting LGBTQ2S+ people.”

 Second, to the best of our knowledge, there has been little to no enforcement of bans. Crime statistics are not yet available, though these could be reported in the future (e.g., by Statistics Canada). The absence of these data further motivates community surveys like the one we conducted. We have added the following line to the Introduction (see page 5): “Moreover, official data on enforcement of bans are absent, and many who experience CTP may choose not to disclose their experiences owing to feelings of shame.”

 Third, we have included a list of municipalities with bans in Table 2. We have added detail regarding the typical mechanism of municipal bans on page 4: “Municipal bans typically operate through prohibition of CTP under bylaws governing the provision of business licenses.”

 Fourth, we have included these in the limitations section on page 14: “Fifth, it was not possible to determine whether the reports were mutually exclusive. This means that the same practice could have been reported multiple times, potentially overemphasizing the prevalence of CTPs in a particular province or territory. Sixth, conclusions about certain regions, such as Prince Edward Island, cannot be reached because they were excluded from the analysis due to having fewer than five reports of CTPs. This exclusion could lead to an incomplete estimation of the prevalence and distribution of CTPs across Canada.”

 Fifth, we have added why SN2019 did not find geographic differences and what the possible limitations of that study could be compared to ours. This new addition can be found on page 13: “On the other hand, the SN2019 study did not find a difference in the prevalence of CTPs across provinces and territories in Canada. The difference in findings between SN2019 and our study may be due to differences in survey methodologies or sample sizes, as our study used a more targeted approach to recruit participants with direct or indirect experiences of CTPs.”
